# Iron Nutrition of Pre-Schoolers in High-Income Countries: A Review

**DOI:** 10.3390/nu15112616

**Published:** 2023-06-02

**Authors:** Linda A. Atkins, Alison C. Spence, Ewa A. Szymlek-Gay

**Affiliations:** Institute for Physical Activity and Nutrition (IPAN), School of Exercise and Nutrition Sciences, Deakin University, Geelong, VIC 3220, Australia; a.spence@deakin.edu.au (A.C.S.); ewa.szymlekgay@deakin.edu.au (E.A.S.-G.)

**Keywords:** iron intake, haem iron, pre-schooler, dietary pattern, eating occasion, bioavailability, iron absorption algorithm

## Abstract

Pre-schoolers are vulnerable to iron deficiency, which, in high-resource countries, is mainly caused by suboptimal or poorly absorbable iron intakes. This review examines the prevalence of inadequate iron intakes and status, and the non-dietary factors associated with these, among children aged between 2 and 5 years within high-income countries. It then considers the quality of the pre-schooler diet in terms of dietary factors, dietary patterns, and iron intakes. Additionally, it discusses the assessment of iron bioavailability and examines the various methods used to estimate the amount of absorbable iron in pre-schooler diets. Knowledge of the adequacy of iron intakes and bioavailability of iron intakes, and dietary patterns associated with iron intakes can facilitate the design and implementation of effectively targeted community-based intervention studies to improve iron intakes and iron bioavailability to minimise the risk of iron deficiency.

## 1. Introduction

It is estimated that, globally, almost one-quarter of the world’s population is affected by iron deficiency, with children under 5 years of age among the most vulnerable [1,2]. The pre-school age (2–5 years) is a period where nutritional requirements are high, but dietary recommendations are often not met [3,4,5,6]. During these years, pre-schoolers gain external influence on food preference, while their autonomy increases, and episodic neophobia often exists [7,8,9]. If iron deficiency develops during the early years, a child’s immediate and long-term health can be severely compromised, especially their immunity and motor and cognitive development [10,11,12]. In high-income countries, inadequate intakes are considered a major cause of iron deficiency [13].

Preventative food-based strategies require knowledge about current intakes and sources of iron. Similarly, data on factors associated with iron intakes and the bioavailability of iron in the diets of children are crucial for the development of preventative strategies. It is important to assess the quality of children’s diets because iron intakes are also likely to track, affecting health in later years [14,15]. Iron nutrition in early childhood is still poorly understood, yet it is of public health importance. Hence, this study aimed to review the existing literature relating to the adequacy of iron nutrition of pre-schoolers within high-resource countries.

Specifically, this paper (1) reviews pre-schooler dietary iron intake and iron status and the non-dietary factors associated with these, (2) reviews pre-schooler diet quality in terms of dietary factors, dietary patterns, and iron intakes, and (3) briefly explains iron absorption, forms of iron and their food sources, dietary modifiers of iron absorption, and the estimation of dietary iron absorption.

## 2. Studies of Iron Nutrition among Pre-Schoolers in High-Resource Countries

A summary of the studies on dietary iron intake and iron status is presented in Table 1. Only studies using random sampling techniques from high-income nations that measured the iron intakes and/or iron status of representative samples of children aged 2–5 years and that were available in English are summarised.

Of the seventeen studies of children aged 2–5 years estimating dietary iron intake [17,18,19,20,22,23,25,28,29,30,31,32,34,35,37,38], most reported prevalence of inadequate intakes, which ranged from <1 to 66% [17,18,19,20,22,23,28,29,32,34,35,38]. The proportion of New Zealand children reported as at risk of inadequate intakes was 66% [32], a considerably higher prevalence than <1% as reported in the US [18], however there are notable differences within the methodology of both studies, particularly with the range of Estimated Average Requirement (EAR) values applied. The US study of Butte et al. assessed the adequacy of iron intakes using national 24-h recall data and an EAR of 3 mg per day [18]. Undertaken in the previous decade, and prior to the establishment of the current EAR of 4 mg per day [39], the New Zealand study assessed iron intakes via weighed food records from a cross-sectional, representative sample against the UK EAR of 5.3 mg per day [32,40]. When the current EAR was subsequently used to assess inadequate iron intake for this cohort, prevalence was closer to 30% [41], which was still much higher than was reported in the US [17,18,23].

Chouraqui et al. applied the cutpoint method to data from food diaries and reported that more than half of the iron intakes of French pre-schoolers aged 2–3 years were below the average requirement of 5 mg per day [19]. For assessing the adequacy of intakes from population data, the cutpoint method is appropriate, but not in all cases [42]. Calculating the proportion of iron intakes below the recommended intake to estimate inadequacy is not a suitable method to use for data of children aged 1–8 years, as the asymmetry of requirements is not taken into account [42]. The appropriate method for estimating adequacy of iron intakes of pre-schoolers is the statistical full probability approach, which accounts for the positively skewed iron requirement distributions of this age group [42].

Accurate estimation of the prevalence of inadequate iron intake requires data from usual intake to avoid the within-person variation among observed intakes [43]. Observed intake data from a limited number of days can be adjusted with statistical programs to yield a distribution with reduced variability, while preserving the shape of the original observed distribution. Prevalence of inadequate intakes of total iron for Australian pre-schoolers using these approaches is 10% [16] and for Belgian pre-schoolers is 35–55% [22].

Criteria used to assess biomarkers of iron depletion also differ within the literature. Among the nine studies reporting on iron status of pre-schoolers [13,17,18,21,24,26,33,36,38], serum ferritin (SF) cut-off concentrations of either <10 or <12 μg/L have been applied to indicate depleted iron stores [13,17,21,26,33,36]. Prevalence of depleted iron stores ranged from 7.4% of pre-schoolers in the US [17] to 31% in the UK [13], both using an SF cut-off of 12 μg/L. Prevalence of iron deficient erythropoiesis (IDE) and iron deficiency anaemia (IDA) ranged between 3% in the US and 9% in Iceland, and <1% in the US and 4.3% in New Zealand, respectively [21,26,33]. 

## 3. Non-Dietary Factors Associated with Iron Status or Iron Intakes of Pre-Schoolers

There are multiple non-dietary and dietary factors that can potentially affect iron intake and status both independently and in combination [13,44,45]. Non-dietary factors include socio-demographic characteristics, such as age, sex, family structure, ethnicity, and socio-economic status. Dietary factors associated with iron intakes and iron status are discussed in the following section.

As young children are entirely dependent upon their caregivers, investigations are often focused on the socio-demographic circumstances of the family. Few non-dietary factors associated with pre-schooler iron intakes have been identified. Inverse associations have been reported between pre-schooler iron intakes and age [16] and maternal education [22], while greater iron intakes have been observed among female pre-schoolers [16], and those from families with two or fewer children [22].

In studies where biochemical data have been available for young children, associations between socio-demographic characteristics and iron status have been observed [13,46]. Iron deficiency is more likely to occur in younger children from disadvantaged families, and for children aged 2–5 years, lower concentrations of SF have been observed among males and pre-schoolers of low-income households [13].

## 4. Dietary Quality and Iron Nutrition among Pre-Schoolers

Non-dietary and dietary factors can interact in young children, and the additive effect can double or treble the risk of iron deficiency [47]. A range of dietary factors exists that can influence iron nutrition, either indirectly, such as modifiers of non-haem iron absorption, or directly in terms of the balance of the total diet. The presence and frequency of the consumption of certain foods in the diet appear to influence the risk of iron deficiency. For example, the prevalence of iron deficiency is lower for children who consume iron-fortified formula [45,46], and a positive association is evident between iron status and the consumption of meat and fruit [13]. Additionally, the inverse association between iron status and the type and quantity of milk and milk products has been well documented [13,33,44,45,46,48,49,50,51].

Unmodified cow’s milk is low in iron and potentially displaces iron-rich foods from the diet [13]. Daily cow’s milk consumption of more than 500 mL has been independently associated with low iron status in toddlers [33]. Studies have also reported an inverse association between iron status and the daily consumption of more than 400 mL of cow’s milk or milk products for pre-schoolers [13,45,50]. In fact, pre-schoolers who consume more than 600 mL of cow’s milk per day are almost four times more likely to be iron depleted [51].

### 4.1. Dietary Factors and Pre-Schooler Iron Intakes

Dietary factors influencing the iron intakes of pre-schoolers across Europe include iron-fortified formula [52] and cereal consumption [20,45]. The UK [20] and German [52] nation-wide studies assessed dietary records from multiple days of younger pre-schoolers (aged <3 years). Although iron from formula and cereal is less bioavailable than from foods containing haem iron, their positive association with iron intakes is not surprising given their prominence in the diets of children. For Australian pre-schoolers, sources of haem iron contribute only 8% to dietary iron intakes [53]. Australian children under 8 years of age obtain over 50% of their iron intake from cereals and cereal-based products [3]. These food groups include iron-fortified breakfast cereals (often high in added sugars) and discretionary foods, such as cakes and biscuits.

### 4.2. Dietary Patterns and Iron Intakes of Pre-Schoolers

Dietary pattern analysis allows for a greater understanding of interactions that may occur between food components [54], and due to the broader focus of dietary intake, may offer great insight into disease risk [55]. As dietary pattern analysis is used to examine diets in relation to disease risk and prevention [56], a greater understanding can be achieved from dietary pattern analysis, in terms of iron nutrition.

Despite the importance of understanding the nutrient intake profiles of dietary patterns [57], only a small number of studies have reported on the associations between the dietary patterns and nutrient intakes of children aged 2–5 years in high-resource countries [4,53,58,59,60,61,62,63,64,65,66,67,68]. Of the diet quality studies published in English and focused on pre-school nutrient intakes, twelve have reported patterns associated with iron intakes (Table 2) [53,58,59,60,61,62,63,64,65,66,67,68]. These studies have implemented a variety of approaches, including three that used PCA [53,58,64], they were from a variety of locations (South Korea, the Netherlands, UK, USA, Australia, and Greece), and they reported varied results. Iron intake has been positively associated with pre-schooler diets characterised by healthier foods (such as fruit, fish, rice, and vegetables) [58,64], fruit juice [60], ‘good’ diets [61], and non-haem and non-iron fortified foods (such as cheese and bread) [53]. In addition, it has been inversely associated with patterns dominated by confectionery and convenience foods [58,59] and snack frequency [66]. For South Korean children, where cluster analysis identified two patterns for foods consumed outside of the home, the index of nutritional quality for iron was lower for a mixed pattern than for a rice-centred pattern [67].

## 5. Iron Absorption

To further examine the quality of pre-schooler iron intakes, consideration of the interactions between the enhancing and inhibitory effects of dietary factors can provide an understanding of the extent to which pre-schooler iron intakes are absorbable. It is necessary to absorb adequate iron to support growth [69].

While iron intakes have not been shown to directly predict iron absorption in young children in some studies [70], they do influence iron stores [71], which, in turn, are a good predictor of iron absorption in young children [70,72]. An inverse relationship exists between absorption and iron stores [73]; when iron stores are sufficient, less iron is absorbed. This regulatory feedback is instrumental in iron homeostasis [74].

### 5.1. Forms of Iron, Food Sources, and Absorption of Iron

In addition to iron stores [75], both the content and the bioavailability of the two forms of iron present in foods, haem and non-haem, also determine the amount of dietary iron absorbed [76]. Haem iron is found only in animal tissue and is readily absorbed; animal tissue itself also promotes the absorption of non-haem iron [77].

The mean absorption of haem iron from a meat-containing meal has been estimated to be 25% [78]. In iron-deficient individuals, this can increase to 40%, while in those who are iron-sufficient, only 10% of haem iron is usually absorbed [79]. Among meat-eating populations, it is estimated that only 10–15% of total iron consumed is haem iron, however, due to its efficient absorption, haem iron contributes over 40% of the total absorbed iron [80].

Non-haem iron is less bioavailable than haem iron [80] and is found in meat and animal- (e.g., milk, eggs) and plant-based foods, including iron-fortified products [43]. The absorption of non-haem iron occurs early in digestion, as iron solubility is favoured by the low pH of the duodenum [81]. The formation of insoluble ferric complexes beyond the duodenum most likely reduces the bioavailability further [81]. The absorption of non-haem iron is influenced by accompanying food components [82], which are discussed in the following section.

### 5.2. Dietary Modifiers of Iron Absorption

The presence of certain factors within a meal can modify iron absorption [82,83]. Ascorbic acid and animal tissue are known enhancers of iron absorption [76], while potential inhibitors include polyphenols and phytates, particularly myo-inositol hexakisphosphate, or IP6, as well as calcium and soy protein [43].

#### 5.2.1. Ascorbic Acid

Vitamin C is widely available in fruits and vegetables and is referred to as ascorbic acid when in its functional, in vivo form. Ascorbic acid reduces ferric iron (Fe^3+^) to the more soluble ferrous iron (Fe^2+^), enhancing its absorption [84]. The addition of 50 mg ascorbic acid (equivalent to approximately 100 g of fresh orange) to foods, including iron-fortified foods, can improve iron absorption by up to 164% [85,86,87] and can considerably counteract the inhibitory effects of phytates and polyphenols [88]. Iron inhibition from 25 mg phytate phosphorus (as can be found in an 80 g wheat roll) [89] can potentially be negated with 80 mg ascorbic acid [88]. The promotion of iron absorption by ascorbic acid is dose-dependent [90], and is more pronounced in the presence of phytates or polyphenols [76].

#### 5.2.2. Animal Tissue

Originally reported in 1968 [83], the observation of the effect of meat on non-haem iron absorption has since been supported by further studies [91,92]. Commonly referred to as the “meat, fish, poultry (MFP) factor” [93], “meat factor” [73,94], or “meat effect” [95], the presence of animal tissue promotes the absorption of both non-haem iron [73,91] and haem iron [94].

This enhancing effect has been attributed to the gastric acid secretion stimulated by meat digestion and a lowered gastric pH, which maintains the solubility of iron [95]. The role of cysteine-containing peptides has also been investigated [96]. In a study that isolated the effect of protein source on iron absorption between soy and beef meals of equal protein and non-haem content [92], the beef was found to increase non-haem iron absorption at almost twice the rate of the soy protein. Although the exact mechanism is unclear, the presence of animal proteins is believed to be the driving force enhancing iron absorption [97].

#### 5.2.3. Polyphenols

Polyphenol compounds, found naturally in various vegetables, fruit, cereals, spices, tea, coffee, and wine bind with non-haem iron, inhibiting its absorption [76]. Groups of phenolic compounds are classified by their chemical structure as polyphenolic amides, phenolic acids, flavonoids, and other polyphenols, including ellagic acid, rosmarinic acid, resveratrols, and hydrolysable tannins [98,99]. Tannins present in tea have been known to inhibit the absorption of iron from food sources, such as bread and rice [100]. The effect on iron absorption by different polyphenols has been researched by multiple groups [101,102]. Brune et al. observed an inhibiting effect of gallic acid and tannic acid, which was dose-dependent [101]. Hurrell et al. observed a potential inhibiting effect on non-haem iron absorption by all major food polyphenols, especially black tea [102].

#### 5.2.4. Phytate

Phytate (phytic acid), a storage form of phosphorous, and present in seeds, nuts, legumes, and grains, inhibits iron absorption by strongly binding ferrous iron [87,103]. While high phytate intake is commonly observed in low-and middle-income nations, efforts to increase fibre consumption in high-income countries may also inadvertently increase phytate consumption [88]. The inhibitory effect of phytate is dose-dependent, with marked effects on iron absorption observed when as little as 2 mg was added to 40 g wheat rolls [88]. However, there are methods to reduce the phytate content of foods, which include soaking, fermentation, germination, and the addition of a phytase enzyme.

#### 5.2.5. Calcium and Soy Protein

There are other factors in food that can inhibit iron absorption. Supplement and single-meal studies have suggested that calcium has an inhibitory effect on haem iron absorption [43,94,104], but little evidence exists on whole diets [76,105]. Significant reductions in iron absorption due to the presence of soy protein have been reported by some [80,106] but not all studies [92,107]. It has been proposed that it is not the presence of soy in a meal, but the substitution of meat for soy that causes reduced absorption of iron [107]. It has also been suggested that it may depend on the type of soy protein [92] and that the fermentation of soy degrades the protein and enhances its bioavailability [84].

### 5.3. Determining Iron Absorption via Isotope Absorption Studies

The inhibitory/enhancing effect of food components on iron absorption has been assessed with the use of isotope absorption studies. Radioactive or stable isotope absorption studies allow for the direct measurement of human iron absorption [69,108]. Although these studies are specific and reliable, they are costly and time-consuming [108]. Isotope studies provide iron absorption values for the particular meal studied but not necessarily for alternative combinations of meals consumed. Additionally, potential ethical implications associated with human experimentation, the use of radioactive isotopes, and the need for specialised technology limit their use [108]. Only stable isotopes can be ethically used in children [109]. As isotope studies are often not practical, especially at a population level, algorithms are universally accepted as a cost-effective alternative to estimate iron bioavailability [108].

### 5.4. Estimation of Iron Absorption via Algorithms

Over the years, many algorithms have been developed to quantify the impact of mechanisms involved in iron balance [73,76,110,111,112,113,114,115,116,117,118,119,120,121], and some of these algorithms have been compared to each other and to biochemical data to determine their accuracy and validity for predicting iron bioavailability [93,108]. The algorithms take into consideration the relationship between iron intake from all sources and body loss and storage and account for various modifying dietary factors. However, most of these have been developed based on data from adult populations, which may not be applicable to the diets of young children. Only two of the algorithms have been developed for study populations that include children [115,120]. Another consideration is that most algorithms have been developed from single-meal tests, which may exaggerate the effect a dietary factor would have over several days [43,119,122]. The iron absorption from single-meal tests can also differ from that from whole diets [119,123].

Algorithms are also restricted in their ability to estimate iron absorption from meals containing both inhibitors and enhancers because of the interactions that occur between the modifying components [43], and they offer only approximate calculations compared to in vivo experiments [108]. Despite these limitations, algorithms allow for the efficiency of iron absorption to be estimated from different diets and for the estimation of the effects of dietary modification on iron absorption at a population level. The application of algorithms to estimate iron bioavailability in population studies is necessary because direct measurement is often not a feasible option.

The selection of an algorithm to estimate iron absorption in a population may depend on the age, size, and location of the study population, as well as the type of dietary and/or biochemical data available. The use of adult-based algorithms to estimate iron absorption of pre-schooler diets may not be ideal, yet few algorithms exist that may be suitable for children [115,120]. The algorithms of Murphy et al. and Tseng et al. were developed specifically for children’s diets; thus far, they have been applied to a small number of studies in low- and middle-income countries [115,120,124].

## 6. Summary

The prevalence of inadequate intakes of iron among pre-schoolers in high-income countries varies between <1 and 66%, while the prevalence of depleted iron stores ranges between 7.4 and 31%. Age, sex, maternal education, and household size are non-dietary factors known to influence pre-schooler iron intakes. Greater iron intakes are more likely to be observed among female pre-schoolers and those from smaller families, while child-age and maternal education are inversely associated with pre-schooler iron intakes. Dietary factors that are positively associated with pre-schooler iron intakes include the consumption of iron-fortified formula and cereal, and dietary patterns characterised by various combinations of core foods.

Understanding the adequacy and bioavailability of pre-schooler iron intakes can facilitate the design and implementation of effectively targeted community-based intervention studies to improve iron intakes, particularly of bioavailable iron, and minimise the risk and consequence of iron deficiency. Further research is required to estimate the adequacy of absorbable iron among pre-schoolers. A feasible approach to this is via the application of appropriate algorithms.

## Figures and Tables

**Table 1:** Studies of intake and status of iron among pre-schoolers in high-resource countries.

Source, Location, Year	Subjects	Methodology	Iron Intake (mg/d)	Prevalence of Inadequate Intake (%)	Biomarkers of Iron Deficiency	Prevalence of Iron Deficiency (%)
Atkins et al. [16], Australia, 2011–2012	2–5 y *n* = 783	Study Design: Nationally representative cross-sectional survey data from the National Nutrition and Physical Activity Survey component of the Australian Health Survey. Data: 2 × 24 h recall via automated multiple-pass method. Usual intake estimated via PC-SIDE. Full probability method used to estimate prevalence of inadequacy.	Mean (SD): 7.9 (1.9)	10.1	NA	NA
Bailey et al., 2021 [17], USA, 2001–2016	1–6 y *n* = 9848	Study Design: Nationally representative cross-sectional survey data from the National Health and Nutrition Examination Survey (NHANES) 2001–2016. Data: 2 × 24 h recall via automated multiple-pass method. Usual intake estimated via NCI method. EAR cutpoint method used to estimate prevalence of inadequacy (≤3 y: 3 mg/d; ≥4 y: 4.1 mg/d). Blood samples collected.	Mean (SE): 11.6 (0.1)	1.1	ID: SF < 12 µg/L ID: SF < 10 µg/L Anaemia: Hb < 110 g/L	1–6 y: 7.4 1–3 y: 10.7 4–6 y: 3.7 1–6 y: 4.6 1–6 y: 2.5 1–3 y: 3.6 4–6 y: 1.6
Butte et al., 2010 [18], USA, 2008–2009	24–47 mo *n* = 1461	Study Design: Cross-sectional, national survey data from the Feeding Infants and Toddlers Study (FITS) 2008. Data: Multiple pass 24 h recall via telephone. A second recall was completed by a random subsample (*n* = 701). Usual intake estimated via PC-SIDE. EAR cutpoint method used to estimate prevalence of inadequacy (3 mg/d).	Mean (SE): 12.9 (0.17)	<1	NA	NA
Chouraqui et al. 2020 [19], France, 2013	24–29 mo: *n* = 125 30–35 mo: *n* = 81	Study Design: Cross-sectional, national survey data from Nutri-Bebe study 2013. Data: Food diaries from 3 non-consecutive days, including 1 weekend day at 2 personal interviews. EAR cutpoint method used to estimate prevalence of inadequacy (5 mg/d).	Mean (SD): 24–29 mo: 5.9 (2.2) 30–35 mo: 7.0 (4.3)	>50	NA	NA
Gibson and Sidnell 2014 [20], UK, 2008–2011	18–35 mo *n* = 185	Study Design: Cross-sectional data from the National Diet and Nutrition Survey (2008–11). Data: 4 d food diary. EAR cutpoint method used to estimate prevalence of inadequacy (5.3 mg/d).	Mean: 6.4 (SD or SEM not reported)	31	NA	NA
Gunnarsson et al., 2004 [21], Iceland	25–36 mo *n* = 71	Study Design: Cross-sectional national survey of randomly selected 2 y old children. Data: Fasting blood samples collected.	NA	NA	ID: SF < 12 µg/L IDE: SF < 12 µg/L and MCV < 74 fL IDA: Hb < 105 g/L, SF < 12 µg/L and MCV < 74 fL	27 9 1.4
Huybrechts et al., 2012 [22], Belgium, 2002–2003	2.5–6.5 y *n* = 661	Study Design: Random cluster sampling design at the level of schools, stratified by province and age for Flanders pre-school dietary survey. Data: Estimated dietary records completed by parents for 3 consecutive days. Usual intake estimated via C-SIDE. In absence of national EAR, used US values to determine prevalence of inadequacy via the full probability approach.	Boys (*n* = 338): Mean (SD): 7.4 (2.3) Girls (*n* = 323): Mean (SD): 6.7 (2.2)	<4 y: 35 ≥4 y: 55	NA	NA
Jun et al., 2018 [23], USA, 2015–2016	24–47.9 mo *n* = 596	Study Design: Cross-sectional, national survey, Feeding Infants and Toddlers Study (FITS) 2016. Subjects categorised as participants of the Special Supplemental Nutrition Program for Women, Infants, and Children (WIC, *n* = 161), lower- income non-participants (LINP, *n* = 135), or higher-income non-participants (HINP, *n* = 300). Data: Multiple pass 24 h recall via telephone, second recall for random subsample (*n* = 799). Usual intake estimated via NCI method. EAR cutpoint method used to estimate prevalence of inadequacy (≤3 y: 3 mg/d; ≥4 y: 4.1 mg/d).	Mean (SE): WIC: 11 (0.05) LINP: 10 (0.4) HINP: 9.5 (0.3)	1.7 3.7 2.6	NA	NA
Karr et al. 1996 [24], Australia, 1992–1994	24–62 mo *n* = 496	Study Design: Cross-sectional. Cluster sampling within census collection districts and household. Data: Venous blood samples.	NA	NA	ID: SF < 12 µg/L IDE: SF < 12 µg/L and MCV < 70 fL or ZPP > 80 µmol/mol haem	10.5 2.8
Kyttala et al. 2010 [25], Finland, 2003–2005	2 y: *n* = 230 3 y: *n* = 471 4 y: *n* = 554	Study Design: Cross-sectional sample of participants of Type 1 Diabetes Prediction and Prevention (DIPP) birth cohort study. Data: Dietary records for 3 consecutive days, including a weekend day close to the child’s birthday were completed by parents and child-care staff.	Mean (SD): 2 y: 5.9 (2.2) 3 y: 7.0 (4.3) 4 y: 7.5 (3.3)	NA	NA	NA
Looker et al., 1997 [26], USA, 1988–1994	1–2 y *n* = 1339 3–5 y *n* = 2334	Study Design: Nationally representative cross-sectional survey data from NHANES III. Data: Venous blood samples.	NA	NA	IDE: 2 of 3 from FEP > 80 µg/dL of red blood cells; TS < 10% for 1–2 y, <12% for 3–5 y; SF < 10 µg/L IDA: Hb < 110 g/L and IDE	1–2 y: 9 3–5 y: 3 1–2 y: 3 3–5 y: <1
Mackerras et al., 2004 [27], Australia, 1995	2–4 y *n* = 1085	Study Design: Nationally representative cross-sectional data from 1995 National Survey of Lead in Children (NSLIC). Data: Blood samples were collected from children in the home by skilled paediatric blood collectors.	NA	NA	WHO criteria: 6–59 mo: haematocrit < 33% US criteria: <2 y: haematrocrit <32.9% <5 y: haematrocrit ≤33%	2 3.3
Magarey and Bannerman 2003 [28], Australia, 1995–1996	2–18 y *n* = 3007	Study Design: Nationally representative cross-sectional data from 1995 National Nutrition Survey and comparison to 1985 national survey data which focused on prevalence of poor intakes (<0.7 RDI of 6–8 mg/d). Data: 24 h recall (via proxy for children < 5 y).	Not reported	Males 2–3 y: 4.4 4–7 y: 0.2 Females 2–3 y: 11.0 4–7 y: 2.8	NA	NA
McAfee et al. 2012 [29], Seychelles, 2006	5 y *n* = 229	Study Design: Cross-sectional analysis of longitudinal data. Participants of the Seychelles Child Development Nutrition Study (SCDNS) followed up at 5 y. Data: 4 d WDR. Usual intake estimated. EAR cutpoint method used to estimate prevalence of inadequacy (4.7 mg/d).	Mean (SD): 6.3 (1.6)	Males: 8 Females: 6	NA	NA
Radcliffe et al. 2002 [30], Australia, 1995–1996	2–5 y *n* = 793	Study Design: Cross-sectional data from 1995 National Nutrition Survey. Data: 24 h recall (via proxy for children < 5 y).	2–3 y (*n* = 383): Mean 7.8 (95% CI: 7.5-8.1) 4–5 y (*n* = 410): Mean 8.9 (95% CI: 8.5–9.2)	NA	NA	NA
Sichert-Hellert et al., 2001 [31], Germany, 1985–2000	2–<4 y: *n* = 916 4–<7 y: *n* = 1237	Study Design: Cross-sectional data from Dortmund Nutritional and Anthropometric Longitudinally Designed Study (DONALD). Data: 3 d WDR.	Mean (SD): 2–<4 y: 5.8 (1.6) 4–<7 y: 7.3 (1.9)	NA	NA	NA
Soh et al., 2002 [32], New Zealand, 1998–1999	12–24 mo *n* = 184	Study Design: Cross-sectional survey with proportionate cluster sampling. Data: Non-consecutive 3 d WDR. Usual intake estimated via C-SIDE. EAR cutpoint method used to estimate prevalence of inadequacy (5.3 mg/d).	Boys: Median (IQR): 4.4 (3.3, 5.3) Girls: Median (IQR): 4.8 (3.4, 6.6)	66	NA	NA
Soh et al., 2004 [33], New Zealand, 1998–1999	12–24 mo *n* = 169	Study Design: Cross-sectional survey with proportionate cluster sampling. Data: Non-fasting venipuncture blood sample.	NA	NA	ID: SF ≤ 10 µg/L, no IDE or IDA SF ≤ 12 µg/L, no IDE or IDA IDE: Hb ≥ 110 g/L and 2 of 3 from MCV ≤ 73 fL, ZPP ≥ 70 µmol/mol haem and/or SF ≤ 10 µg/L or SF ≤ 12 µg/L IDA: Hb < 110 g/L and 2 of 3 from MCV ≤ 73 fL, ZPP ≥ 70 µmol/mol haem and/or SF ≤ 10 µg/L or SF ≤ 12 µg/L	12.6 18.6 4.3 5.6 3.5 4.3
Thane et al., 2000 [13], UK, 1992–1993	1.5–4.5 y *n* = 1003	Study Design: Cross-sectional data from National Diet and Nutrition Survey. Data: 4 d WDR, including 2 weekend days and biochemical assessments.	NA	NA	Hb < 110 g/L SF < 10 µg/L SF < 12 µg/L Both low Hb (<110 g/L) and SF (<10 µg/L)	8 20 31 3.4
Walton et al., 2017 [34], Ireland, 2010–2011	12–59 mo *n* = 500	Study Design: Cross-sectional data from Irish National Pre-School Nutrition Survey. Data: 4 d WDR, including at least 1 weekend day. Usual intake estimated via NCI method. EAR cutpoint method used to estimate inadequacy (5.0 mg/d).	Mean (SD): 7.3 (0.8) (total *n* = 500)	9.4 (total *n* = 379)	NA	NA
Webb et al., 2008 [35], Australia, 1997–2000	16–24 mo *n* = 429	Study Design: Cross-sectional data from Childhood Asthma Prevention Study (CAPS). Data: 3 d WDR, including 1 weekend day. EAR cutpoint method used to estimate inadequacy (4.0 mg/d).	Mean (SEM): 5.8 (0.23)	23.3	NA	NA
Weiler et al., 2015 [36], Canada, 2010–2011	2–5 y *n* = 430	Study Design: Cross-sectional sample of healthy full-term children from daycare centres. Data: Diet records by dietitian followed by 24 h recall by parent. A nonfasted capillary blood sample was collected via finger lance at daycare.	Mean (SD): 10.3 (4.9)	NA	ID: SF < 12 µg/L for < 5 y, or <15 µg/L for ≥ 5 y with CRP < 5 mg/L IDA: low SF and Hb < 110 g/L for <5 y, or < 115 g/L for ≥ 5 y	% (95% CI): 16.5 (13.0−20.0) 3.0 (1.4−4.6)
Weker et al., 2011 [37], Poland, 2010	13–36 mo *n* = 400	Study Design: A representative nation-wide sample of children were recruited via random selection of personal identity number. Data: 3 d diet records, including a weekend day.	Mean (SD): 8.5 (3.0)	NA	NA	NA
Zhou et al., 2012 [38], Australia, 2005–2007	>24–65 mo *n* = 206	Study Design: Cross-sectional survey. Data: 3 d WDR, including 1 weekend day. Maternal recall used for breastfeeding duration. EAR cutpoint method used to estimate inadequacy (4.0 mg/d). Collection of non-fasting blood samples.	Median (IQR) >2–3 y: 6.6 (5.0, 7.7) >3–4 y: 6.4 (5.1, 7.9) >4–5 y: 7.6 (6.2, 8.5)	>2–3 y: 12 >3–4 y: 7 >4–5 y: 0	ID: SF < 10 µg/L IDA: SF < 10 µg/L and Hb < 105 g/L for ≤ 24 mo, or Hb < 115 g/L for > 24 mo	>2–3 y: 5 >3–4 y: 3 >4–5 y: 3 >2–3 y: 3 >3–4 y: 0 >4–5 y: 0

Abbreviations: CRP: C-reactive protein, d: day, EAR: estimated average requirement, FEP: free erythrocyte protoporphyrin, Hb: haemoglobin, h: hour, ID: iron depletion, IDA: iron deficiency anaemia, IDE: iron deficient erythropoiesis, IQR: interquartile range, MCV: mean corpuscular volume, mo: month, NA: not assessed, NCI: National Cancer Institute, RDI: recommended dietary intake, SD: standard deviation, SEM: standard error, SF: serum ferritin, TS: transferrin saturation, WDR: weighed diet records, y: year, ZPP: erythrocyte zinc protoporphyrin.

**Table 2:** Studies of pre-schooler dietary patterns associated with iron intakes.

Source, Location	Subjects	Study Design, Name, and Dietary Assessment	Diet Pattern Approach	Patterns or Clusters Identified	Associations Reported
Atkins et al., 2021 [53], Australia	2–5 y	Cross-sectional 2011-12 National Nutrition and Physical Activity Survey component of the Australian Health Survey 2 × 24 h recalls	PCA	1. Positive loadings for cheese, breads, fats and oils, and water. Negative loading for milk. 2. Positive loadings for yoghurt, breakfast cereals (non-iron- fortified), grains, and other cereals and legumes. Negative loading for discretionary snacks and beverages. 3. Positive loadings for red meat, iron-fortified fruit and vegetable products, and sauces and spreads. Negative loadings for fruit, mixed vegetable dishes, and yoghurt.	Pattern 1 was positively associated with dietary iron intakes. Pattern 3 was negatively associated with both dietary and non-haem iron intakes.
Cribb et al., 2013 [64], UK	3–9 y 3 y: *n* = 10023 4 y: *n* = 9550	Ongoing longitudinal Avon Longitudinal Study of Parents and Children (ALSPAC) Parents completed FFQ when children were aged 3, 4, 7, and 9 y	PCA	1. Processed: high fat/sugar processed foods. 2. Health conscious: vegetarian style foods, salad, rice, pasta, fruit, and fish. 3. Traditional: meat, potatoes, and vegetables.	Positive correlation between health- conscious pattern and iron intake.
Golley et al., 2011 [62], Australia	4–16 y *n* = 3416	Cross-sectional 2007 Australian Children’s Nutrition and Physical Activity Survey (CNPAS) 2 × 24 h recalls	Assessed adherence to dietary guidelines using DGI-CA. DGI-CA is the sum of 11 components. Scores closer to 100 reflect greatest adherence.	NA	Higher nutrient (including iron) intakes were positively associated with higher DGI-CA scores. Younger children had higher scores. For younger children (4–7 y), scores were positively associated with household education.
Ju et al., 2021 [67], South Korea	3–11 y *n* = 306	Cross-sectional 2016–2018 Korea National Health and Nutrition Examination Survey 24 h recall	Cluster analysis	1. Rice-centred. 2. Mixed.	Index for nutritional quality for iron was lower for the mixed pattern.
Kranz et al., 2006 [63], USA	2–5 y *n* = 5437	Cross-sectional 1994–98 US Department of Agriculture Continuing Survey of Food Intakes by Individuals (CSFII) 2 × 24 h recalls	Assessed whether index scores were associated with differences in food or nutrient intake. Developed a food-based diet score specifically for 2–5 y American children.	NA	Children in the lowest quartile of total index points scored lower in all index components, including iron, compared to children in the highest quartile.
LaRowe et al., 2007 [60], USA	2–5 y *n* = 541	Cross-sectional 2001–02 National Health and Nutrition Examination Survey (NHANES) 24 h recall	Cluster analysis 8 beverage groups formed according to intake of high-fat milk, reduced-fat milk, fruit juice, soda, diet soda, sweet beverages, coffee and tea, water.	1. Mix/light drinker. 2. High-fat milk drinker. 3. Water drinker. 4. Fruit juice drinker.	Iron intakes differed by cluster. Fruit juice cluster had highest iron intake.
Manios et al., 2009 [61], Greece	2–5 y *n* = 2287	Cross-sectional 2003-04 Growth, Exercise and Nutrition Epidemiological Study In preSchoolers (GENESIS) Combination of weighed food records, 24 h recalls and food diaries	Assessed whether index scores were associated with differences in food or nutrient intake. Used Healthy Eating Index (HEI).	Poor diet = score < 5 Needs improvement diet = score 5.1–8 Good diet = score > 8	Higher nutrient (including iron) intakes strongly correlated with higher HEI scores.
Mitsopoulou et al., 2019 [66], Greece, 20	1–19 y *n* = 598	Cross-sectional Hellenic National Nutrition and Health Survey 24 h recall	Mean adequacy ratio (MAR) was used to measure diet quality.	1. Breakfast, lunch, dinner, three snacks. 2. Breakfast, lunch, dinner, two snacks. 3. Breakfast, lunch, dinner, one snack. 4. Breakfast, lunch, dinner, no snacks.	No associations of MAR with meals or snacks. For 4–19 y, inverse relationship between snack frequency and iron intakes.
Papanikolaou et al., 2017 [68], USA,		Cross-sectional 2005–10 National Health and Nutrition Examination Survey (NHANES) 24 h recall	Cluster analysis	1. No consumption of main grain groups. 2. Cakes, cookies and pies. 3. Yeast bread and rolls. 4. Cereals. 5. Pasta, cooked cereals and rice. 6. Crackers and salty snacks. 7. Pancakes, waffles and French toast, and other grains. 8. Quick breads.	Energy-adjusted iron intake was greater in all grain clusters compared to no consumption of grains cluster.
Pryer and Rogers 2009 [59], UK	1.5–4.5 y *n* = 1675	Cross-sectional 1992–93 National Diet and Nutrition Survey (NDNS) 4 d WDR	Cluster analysis Used 19 food and beverage groups in analysis.	1. Traditional: cakes, puddings, meat dishes, confectionery, soft drinks. 2. Healthy: whole-grain cereals, low-fat dairy, egg dishes, vegetables, fruit, nuts, and fruit juices. 3. Convenience: refined cereals, cakes, puddings, fat spreads, bacon/ham, candy, French fries/potatoes, and soft drinks.	Traditional cluster had lower nutrient density for most vitamins and minerals for both boys and girls, including for iron.
Shin et al. 2007 [58], South Korea	‘Pre-school’ age mean age: 5.2 y *n* = 1441	Cross-sectional 2001–05 Practical Approach for Better Maternal and Child Nutrition and Health Study Semi-quantitative, 100-item FFQ	PCA FFQ data sorted into 33 nutrient profile-based food groups.	1. Korean healthy: vegetables, seaweed, beans, fruits, milk, and dairy. 2. Animal foods: beef, pork, poultry, fish, and fast foods. 3. Sweets: ice cream, soda, chocolate, and cookies.	Korean healthy pattern scores associated with higher energy- adjusted iron intakes. Sweets pattern scores inversely associated with nutrient intakes, including iron. Iron intakes did not differ across quintiles of animal foods pattern scores.
Voortman et al. 2015 [65], The Netherlands	13 mo: *n* = 3629 25 mo: *n* = 844	Prospective 2001–05 Generation R Study FFQ completed by mothers when child was 1 y and again at 2 y and was validated against 3 d, 24 h recalls conducted by trained nutritionists	Developed a food-based diet score appropriate for use in pre-school children.	NA	Food-based diet score was positively associated with nutrient intake and health-conscious behaviours. After adjustment for age and sex, diet score inversely associated with iron.

Abbreviations: NA: not applicable, d: day, mo: month, y: year, h: hour, DGI-CA: Dietary Guideline Index for Children and Adolescents, FFQ: food frequency questionnaire, PCA: principal component analysis, WDR: weighed diet record.

## Data Availability

No new data were created or analyzed in this study. Data sharing is not applicable to this article.
